# Experiences from consumer reports on psychiatric adverse drug reactions with antidepressant medication: a qualitative study of reports to a consumer association

**DOI:** 10.1186/2050-6511-13-19

**Published:** 2012-12-23

**Authors:** Andreas Vilhelmsson, Tommy Svensson, Anna Meeuwisse, Anders Carlsten

**Affiliations:** 1 Nordic School of Public Health, Gothenburg, Sweden; 2 Department of Behavioural Sciences and Learning, Linkoping University, Linkoping, Sweden; 3 School of Social Work, Lund University, Lund, Sweden; 4 Medical Products Agency, Uppsala, Sweden

## Abstract

**Background:**

The new European pharmacovigilance legislation has been suggested as marking the beginning of a new chapter in drug safety, making patients an important part of pharmacovigilance. In Sweden since 2008 it has been possible for consumers to report adverse drug reactions (ADRs) to the Medical Products Agency (MPA), and these reports are now understood as an increasingly valuable contribution in the monitoring of safety aspects in medicines. Already in 2002 it was possible to report experiences with medicines to the non-profit and independent organization Consumer Association for Medicines and Health (KILEN) through a web-based report form with an opportunity to describe ADR experiences in free text comments. The aim of this study was to qualitatively analyze the free text comments appended to consumer reports on antidepressant medication.

**Methods:**

All reports of suspected adverse reactions regarding antidepressant medications submitted from January 2002 to April 2009 to KILEN’s Internet-based reporting system in Sweden were analyzed according to reported narrative experience(s). Content analysis was used to interpret the content of 181 reports with free text comments.

**Results:**

Three main categories emerged from the analyzed data material: (1) *Experiences of drug treatment* with subcategories (a) *Severe psychiatric adverse reactions*, and (b) *Discontinuation symptoms*; (2) *Lack of communication and* (3) *Trust and distrust*. A majority of the reports to KILEN were from patients experiencing symptoms of mental disturbances (sometimes severe) affecting them in many different ways, especially during discontinuation. Several report included narratives of patients not receiving information of potential ADRs from their doctor, but also that there were no follow-ups of the treatment. Trust was highlighted as especially important and some patients reported losing confidence in their doctor when they were not believed about the suspected ADRs they experienced, making them attempt to discontinue their antidepressant treatment on their own.

**Conclusions:**

The present study indicates that free text comments as often contained in case reports directly submitted by patients can be of value in pharmacovigilance and provide important information on how a drug may affect the person using it and influence his or her personal life.

## Background

The new European pharmacovigilance legislation (Directive 2010/84/EU) (Regulation 1235/2010) [1] that came into force in July 2012 has been suggested as marking the beginning of a new chapter in drug safety [2]. Its purpose is to further accentuate patient influence, and all EU countries are now obliged to introduce patient/consumer reporting to their spontaneous reporting systems, making patients an important part of pharmacovigilance. Since under-reporting by health professionals is a well-recognized problem by the World Health Organization (WHO) [3], the Organization proclaims consumer reporting to be of great importance in safeguarding a pharmacovigilance program that will help each patient to receive optimum therapy, and on a population basis will lead to ensure the acceptance and effectiveness of public health programs [4].

Previous research has indicated that consumer reporting of adverse drug reactions (ADRs) may add value to healthcare professionals’ (HCP) reports by identifying possible new reactions [5-10]. In Sweden since 2008 it has been possible for consumers to submit reports to the Medical Products Agency (MPA), and these reports are now understood as an increasingly valuable contribution in the monitoring of safety aspects in medicines [11]. The MPA also offers the opportunity for the consumer to use free text in describing the reaction(s). However, these descriptions have not previously been subjected to qualitative analysis or been published. In order to strengthen consumer rights within the health care sector, the non-profit and independent organization Consumer Association for Medicines and Health (KILEN) has provided the opportunity for consumers to report their perceptions and experiences of using medicines. KILEN had already established a consumer database in 1997 to collect consumer reports, mainly focusing on benzodiazepines and antidepressants. KILEN was created in 1992, but their co-workers had already a long history of working with pharmaceutical drug dependency when in the 1960s it became clear that the new benzodiazepines were causing dependency and harm. Since 2002 it has also been possible to report suspected ADRs to this organization through a web-based report form with the opportunity to add free text comments of the experience(s) (http://www.kilen.org). Previous research on the KILEN material has shown that consumer reports may contribute important information regarding more serious psychiatric adverse reactions following antidepressant treatment [9]. However, the free text comments were not scrutinized or analyzed. This study, therefore, aimed to qualitatively analyze the free text comments to consumer reports on antidepressant medication.

## Methods

**Figure 1:**
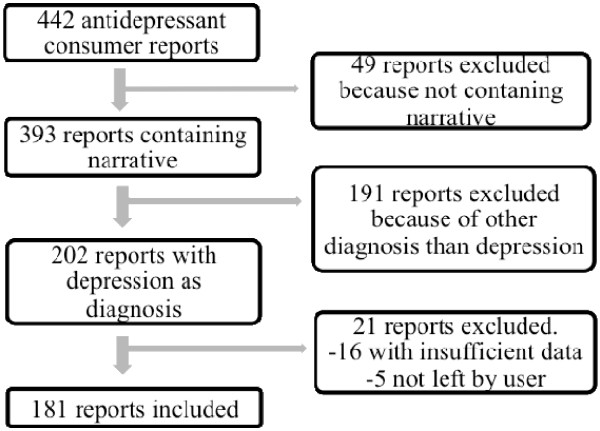
Flow diagram of selected consumer reports to KILEN.

All reports of suspected adverse reactions regarding antidepressant medications submitted from January 2002 to April 2009 to KILEN’s Internet-based reporting system in Sweden were analyzed according to reported narrative experience(s). According to WHO, a side effect is an unintended effect of a pharmaceutical product (occurring at doses normally used by a patient) and where there is a relation to the pharmacological properties of the drug whilst an adverse drug reaction (ADR) is defined as a response to a medicine which is noxious and unintended (occurring at doses normally used in man) and where the response of the patient is of importance, in which individual factors may play an important [3]. Since the reports to KILEN contained individual responses to a drug reported as severe and noxious we decided to use the latter. The KILEN compilation and coding system is described in a previous study [9]. As Figure 1 shows, of 442 individual antidepressant reports, 393 individuals also provided a longer description of the ADR experience as free text (89%). A total of 202 antidepressant reports concerned depression as diagnosis (most reported cause for prescription) and included a narrative of the experiences (46%). A total of 21 reports were excluded since they were reported by someone other than the patient (5) or contained too little information (16). Included in the study, therefore, were 181 reports (41%) with narrative.

The project was approved by the Regional Ethics Review Board in Gothenburg, Sweden (No. 319–10).

### Data analysis

Content analysis was used to interpret the patients’ accounts. Content analysis here refers to a qualitative data reduction and sense-making effort that takes a volume of qualitative material and attempts to identify core consistencies and meanings [12]. The procedure is basically as follows: data are collected and coded by theme or category; the coded data are then analyzed and presented [13]. Creating categories is the core feature of qualitative content analysis and refers to a descriptive level of content; a category often includes a number of sub-categories [14]. All 181 included consumer narratives on depression and antidepressant treatment were read thoroughly several times in order to get an understanding of their meaning. The content of these narratives was then sorted into different main categories and read again, which resulted in subcategories and sometimes new main categories [14]. As Graneheim and Lundman argue, qualitative content analysis interpretation involves a balancing act, where on one hand it is impossible and undesirable for the researcher not to add a particular perspective to the phenomena under study, but on the other hand the researcher must ‘let the text talk’ and not impute meaning that is not there [14]. Therefore, all authors were involved in analyzing the themes that emerged from the data and were also responsible for reading and confirming the analysis. The authors discussed the analyses – the coding, categorization and interpretation of the results – throughout the work process to gain a mutual understanding. This process was also valid for the selection of quotations describing common experiences found within certain categories. This selection was also made in order to problematize the role of the researcher and to avoid missing out on important information or exaggerate specific content.

## Results and discussion

Of the 181 consumer reports included and analyzed, women contributed 135 (75%) and men 38 (21%). The antidepressants most reported for depression as diagnosis were Sertraline (23.8%), Citalopram (23.8%), Venlafaxine (23.2%), Mirtazapine (10.5%), Paroxetine (7.7%), Escitalopram (6.1%) and Fluoxetine (5.0%). As described in Table 1, three main categories emerged from the analysis of the KILEN data: (1) *Experiences of drug treatment* with subcategories (a) *Severe psychiatric adverse reactions*, and (b) *Discontinuation symptoms,* (2) *Lack of communication and* (3) *Trust and distrust*.

**Table 1:** **Categorization of the analyzed components – examples of patients’ statements in the KILEN consumer reports**^**1**^

**Meaning unit**	**Condensed meaning unit**	**Main-category**	**Sub-category**
*“Difficulties concentrating at work, having suicidal thoughts.”*	Patient experienced suicidal thoughts.	Experiences of drug treatment	Severe psychiatric adverse reactions
*“And when the death wish comes, I become so afraid that I start again.”*	Patient experienced feelings of wanting to die when trying to end medication.		Discontinuation symptoms
*“When I first started taking it I received NO [sic!] warnings of adverse drug reactions.”*	Patient received no warnings of side effects from the doctor.	Lack of communication	
*“Decided that after three years of ‘chemical terror’ to discontinue, WITHOUT [sic!] doctor’s approval.”*	Patients decided to end drug treatment without telling the doctor.	Trust and distrust	

^1^ Categorization according to Graneheim & Lundman (2004).

### Experiences of drug treatment

A main category within the KILEN material concerned patients’ experiences of suspected adverse reactions during their treatment with antidepressants. Particularly serious psychiatric adverse symptoms were perceived as something difficult during and after treatment, and especially during discontinuation.

#### Severe psychiatric adverse reactions

A majority of the reports to KILEN were from patients experiencing symptoms of mental disturbances (sometimes severe) affecting them in many different ways. The level of seriousness has also been indicated in the official spontaneous reports made to the Swedish MPA in 2011 where almost half (49.7%) of a total of 597 reports from the general public were deemed serious [11]. Numerous KILEN narratives reported experiencing a kind of blunting affect of the drug, making patients perceiving feeling like ‘zombies’ incapable of having or sharing feelings towards others, even their own family members:

"“I felt completely blunted, that something controlled me so I no longer had contact with my feelings anymore. I became like a zombie who was completely indifferent to everything.” Female, aged 22 years (Sertraline)."

This blunting affect has been described in other research as well, where ‘being-on-SSRIs’ meant an increased distance between takers and their worlds and where previously emotionally close became no more important than ‘anyone else’ [15]. This feeling of distance was sometimes described in the KILEN narratives as a kind of depersonalization or of feeling a sensation of unreality. In a previous study on the KILEN consumer reports, feeling a sensation of unreality was a commonly reported mental disturbance, but was not listed at all as an adverse drug reaction or side effect in the Swedish Physicians’ Desk Reference FASS (building on the Summary of Product Characteristics SPC) [9].

Some of the KILEN reports contained narratives describing an increase in having suicidal thoughts or of such thoughts newly occurring. Despite its seriousness this is not something new. Already in the early 1990s Teicher and colleagues showed that some depressed patients (free of recent serious suicidal ideation) developed intense and violent suicidal preoccupation after 2–7 weeks of SSRI treatment [16], and that antidepressant medication might interfere with normal neuropsychological processes that keep suicidal thoughts from intruding into consciousness [17]. Experiencing suicidal thoughts in everyday life affected patients to a high degree, according to their narratives in the KILEN material:

"“Difficulties concentrating at work, having suicidal thoughts.” Male, aged 45 years (Venlafaxine)."

Obviously it is in self-reported material often uncertain whether suicidal thoughts were evident before medication started or if they were a direct result of the use of antidepressants, but it is nevertheless important to highlight this severe psychiatric derangement, since it may have fatal consequences if ignored. One must not forget that the role of antidepressants in suicide prevention is considered a major public health question given the high prevalence of both depression and depression-related suicidality [18].

Only 16 (8.8%) consumer narratives out of the total 181 included in the study contained positive experiences of antidepressant drug treatment. This may be compared to a Swedish study of antidepressant medication in primary care where almost 67% of responding patients thought that the drug made them feel better (women 70.2% and men 58.5%) [19]. It is also important not forget that the blunting affect of the drug can sometimes be perceived as positive. Previous research has shown that the experience of younger women is that the antidepressant drug enables them to function in daily life activities [20]. The drug is perceived as working by alleviating pain and suffering [15], by suppressing sensations and stopping the person from dwelling on symptoms [21]. Patients whose narratives were positive about drug treatment in the KILEN data often emphasized that the experienced side effect of the antidepressant was a price worth paying, since the prior untreated condition had been much worse:

"“Saved me from total collapse; sure, there have been shorter episodes of depression, but without Paroxetine [Swedish antidepressant brand name – authors’ note] or something like it, I would have been dead or have killed someone.” Male, aged 42 years (Paroxetine)."

#### Discontinuation symptoms

According to KILEN narratives, it was especially during discontinuation of antidepressant drugs that suspected psychiatric adverse reactions were experienced:

"“Discontinuation of antidepressant medication in four days on doctor’s orders, from normal dosage of 50 mg to 25 mg in four days and then nothing. After three days, I experienced a fear of dying and extreme anxiety and had several panic attacks … woke up and found myself standing with a knife towards my stomach on one occasion and on another with the bathrobe belt in my hand. I no longer tolerate any stress at all, which makes me panic and experience dizziness. Have been without antidepressant medication for nine days and experiences hell on earth.” Female, aged 35 years (Sertraline)."

A previous study on the KILEN material indicated that adverse reactions in connection with discontinuation of antidepressant medication was often reported to KILEN but not always mentioned in the Swedish Physicians’ Desk Reference FASS, and when it was mentioned, it was generally regarded as rare [9]. This has also been shown in an evaluation of the UK patient reporting system ‘Yellow Card Scheme’ which identified new ‘serious’ reactions not previously included in the SPC [5]. Research has shown that antidepressant discontinuation in depressed patients can be associated with worsened depression and increased suicidality [22], and that the recurrence risk for depression was much shorter after rapid than after gradual discontinuation of antidepressants [23]. Abrupt discontinuation can also cause a larger increase in the number of adverse discontinuation symptoms [24,25], and a report from the Swedish Council on Technology Assessment in Health Care (SBU) indicated that long-term use of antidepressants (particularly in high dosages) could cause these symptoms if treatment is terminated suddenly or the dosage is substantially reduced [26].

Fear of discontinuation symptoms made some patients afraid of ending their treatment; these patients often continued to take antidepressants, despite the fact that they did not want to be dependent on them. The suspected adverse reactions were not just perceived as unpleasant but also created a fear of stopping taking the antidepressant drug. A concern that the depression might return was one common feeling that was expressed:

"“And when the death wish comes, I become so afraid that I start again.” Female, aged 42 years (Citalopram)."

Since the psychiatric events reported to KILEN often may also occur as a symptom of the illness for which the antidepressant had been prescribed, their (re)appearance may easily suggest that the patient is having a relapse and needs continued treatment. Distinguishing withdrawal from relapse is important but often also very difficult.

### Lack of communication

A second main category concerned the communication between patient and doctor, or rather its absence. Several KILEN narratives concerned patients experiencing a lack of information regarding adverse reactions from their doctor:

"“When I first started taking it, I received NO [sic] warning of adverse drug reactions.” Female, aged 37 years (Venlafaxine)."

If patients are not receiving information about potential adverse reactions, it is indeed worrying. According to treatment recommendations from the Swedish MPA, all patients with depressive symptoms should be met with understanding and empathy and have (their) opportunity to talk about their life situation, feelings and experience, as well as receive information about the disorder and its treatment options; this includes information about the effects of a drug and its potential adverse reactions [27]. However, previous studies have also provided indications that physicians rarely make reference to side effect (in only 16 of 34 consultations) [28], that patients are unaware that suspected adverse reactions may occur [29], and patients may believe that physicians are withholding information about these reactions [30]. There is also the possibility that physicians themselves are not fully aware of the side effects or adverse reactions related to the drugs they prescribe.

Such situations may lead to poor communication between physicians and patients, which may in turn increase risks [31]. Patients need, for example to be warned about, and monitored for, the possibility of increased depressive and suicidal symptoms as part of a discontinuation reaction. This is also of great importance because feelings of uncertainty regarding the safety of a drug are an important reason for nonadherence to treatment [32]. Fear of adverse effects can be a main reason for not accepting SSRI treatment [33]. Some KILEN reports included, for instance, narratives of giving up on antidepressant treatment because of difficult suspected adverse reactions:

"“Moreover, I had nightmares every night, from the moment I fell asleep, and I woke up several times every night soaked in sweat. Unable to get enough sleep, I became more strained. These adverse drug reactions were the major cause for me to give up my antidepressant treatment.” Female, aged 27 years (Sertraline)."

Robust and clear communications between doctor and patient if and when the patients experiences serious adverse effects is therefore of great importance, since serious events like suicidal thoughts or attempts may continue to affect patients’ lives long after the event [34]. However, it is important to acknowledge that communications about the safety of medicines are complex and generally poorly performed, and that differences in risk perception between the public and healthcare professionals exist, which may be a barrier to clear communication [35]. It is therefore vital that we challenge potential communication obstacles in order to provide a safer prescription culture. Patients need a balance of information about antidepressants so that they can decide whether or not to take (or continue to take) drugs prescribed [36]. It is however important to acknowledge that doctors alone are not to be held responsible for this lack of information. For instance, a British study indicated that the patient information leaflet (PIL), that accompanies antidepressant medication, did not always warn of discontinuation symptoms and also presented side effects in a strikingly heterogeneous way, making it difficult for patients to find the required information needed to make an informed choice [36].

In some cases in the KILEN reports patients described not just a lack of communication between doctor and patient, but also that there were no follow-ups of the treatment, and that prescriptions were renewed without a personal contact, for instance, by telephone:

"“The most appalling thing during all these years of medication was that I did not have any contact with somebody who monitored …either when I started, or during, or after I stopped taking my medication.” Female, aged 23 years (Paroxetine)."

The Swedish National Board of Health and Welfare argues that the most important measure to minimize risks is to evaluate the effect of the prescribed antidepressant drug, and on a regular basis review the treatment, so that the patient does not continue to take a drug without clear indication [19]. However, according to a study of antidepressant medication in primary care, the agency found that only 40% of Swedish patients had an appointment for follow-up, and over 60% of these had used antidepressant drugs for over a year [19]. Since antidepressants drugs are relatively often involved in the fatal adverse drug reactions (FADRs) occurring [37] (even if it is a small number of FADRs that occur in total) the extra need for surveillance is worth highlighting. Talking with patients about what influences their decisions about use of medications could affect whether these medications are used optimally [30]. Otherwise, there is risk of poor or inappropriate prescribing, wastage of drugs and unsatisfactory doctor–patient relationships if doctors’ perceptions do not correspond with patients’ preferences [38].

### Trust and distrust

In a third main category, some patients referred to losing trust in their doctor when they perceived that he or she did not take the patient’s story and description of ADRs seriously and/or scaled down their consequences. According to several patient reports, there were sometimes problems of separating the symptoms related to the diagnosed depression from the suspected adverse reactions, where patients almost always interpreted negative experiences as belonging to the drug while the doctor construed them as evidence of the initial depression recurring.

"“She [the doctor – author’s note] ignores discontinuation symptoms from the drug and wants me to start medicating again after I have been through ten days of hell. She believes that my depression has returned… It is totally wrong.” Female, aged 35 years (Sertraline)."

The conflicting accounts between patients and doctors of either drug-induced reactions or initial illness symptoms were especially present during discontinuation. Some patients reported to KILEN that they perceived discontinuation symptoms over a longer period of time, which they perceived as being dismissed by their doctor. Previous research, however, has indicated that discontinuation symptoms sometimes can be severe and prolonged, and may also be mistaken for signs of physical illness or even early signs of relapse, leading to an overestimate of the true effect of the medication [39]. This is unfortunate, since studies have shown that patients can distinguish between suspected adverse reactions and other symptoms [40] and are capable of providing clear descriptions of their experiences and of balancing the benefits and burden of treatment [6]. Some patients reported losing confidence in their doctor when they were not believed about the suspected adverse reactions they experienced:

"“Decided to discontinue after three years of ‘chemical terror’, WITHOUT [sic] doctor’s approval.” Female, aged 41 years (Paroxetine)."

Patients have witnessed dismissive attitudes among health care professionals in other patient reporting systems as well (in this case, the UK ‘Yellow Card Scheme’) [41]. In the KILEN study, the lack of trust towards the treating doctor made some patients attempt to discontinue their antidepressant treatment on their own, sometimes abruptly leading to severe adverse symptoms as a consequence. It is important that mutual trust exists between patients and doctors in order to prevent non-adherence. Doctors with better communication and interpersonal skills are able to detect problems earlier, can prevent medical crisis and expensive intervention, and provide better support to their patients [42].

As indicated in this study, consumer reporting may be an additional way of detecting harmful effects that may have been missed in clinical trials. Patient/consumer reports (as contrasted to those reported by health professionals) come straight from the person who has experienced the drug effect(s); they describe the effect on the person’s life [5,43,44]. It is hoped that the new EU-legislation on pharmacovigilance [1] will help to stimulate the systematic reporting by patients; in this way more attention can also be paid to how drug-related problems are experienced by patients themselves.

### Limitations

The study has several limitations. The KILEN data material was based on spontaneous consumer reports and thereby was selected material, which might have exaggerated a negative view and experience of antidepressant drug treatment. It is therefore unlikely that all views and experiences of antidepressants have been captured. Since it is an Internet-based reporting system, it will most likely benefit younger individuals who are used to handling a computer, but by missing out on the older age groups one risks getting a biased view of patients’ experiences of treatment. A Danish study showed for instance that older female patients with depressive disorder had more negative views of the doctor–patient interaction and of antidepressants [45].

There is also the issue of gender. In a previous study, it was indicated that women reported ADRs to KILEN in a much higher degree: between three and four times more often than men, and sometimes more within certain age groups [9]. This may also be true for the use of these drugs. In this study, women accounted for 75% of the reported narratives. This may be an effect of women, possibly to a higher degree, turning to non-profit organizations for help. It may also be an effect of women tending to have a higher risk of ADRs than men, which increases with age and increased numbers of drugs prescribed [46]. This could also explain women’s over-representation in ADR reporting to KILEN. Furthermore, we do not know how reporting consumers/patients were ‘officially’ diagnosed with depression (ICD-10, DSM IV or other), and we do not know if the reported diagnosis was a ‘valid’ one, since we only have the patients’ own reported experiences to the KILEN website. It is also important to acknowledge that this was only the patients’ perception of ADRs and doctor–patient communication, and we do not have doctors’ perceptions to compare with.

Lastly, there is the question of potential problems with polypharmacy, with an unknown interaction between psychotropic drugs, for instance, different antidepressants and anxiolytics. It is therefore difficult to know if the reported suspected adverse reaction is a result of a specific medication or the combination of a number of medications. As indicated by a Swedish study, the prevalence of polypharmacy, as well as the mean number of dispensed drugs per individual increased, for instance, year-by-year in Sweden from 2005 to 2008 [47].

Despite the limitations of this study, the data are still of value since the material provides unique information of consumer reporting (in Sweden) and patients’ qualitative experiences of antidepressant treatment and suspected adverse reactions.

## Conclusions

The present study indicates that free text comments as often contained in case reports directly submitted by patients can be of value in pharmacovigilance and provide important information on how a drug may affect the person using it and influence his or her personal life.

## Competing interests

The authors declare that they have no competing interests.

## Authors’ contributions

AV, AM and TS were responsible for study concept and design. AV acquired the data. All authors interpreted the data. AV drafted the manuscript, to which all authors contributed. All authors read and approved the final manuscript.

## Disclaimer

The opinions or assertions contained herein are the private views of the authors and are not to be construed as official or reflecting the views of the Medical Products Agency.

## Pre-publication history

The pre-publication history for this paper can be accessed here:

http://www.biomedcentral.com/2050-6511/13/19/prepub
